# The evolution of prompt reaction to adverse ties

**DOI:** 10.1186/1471-2148-8-287

**Published:** 2008-10-17

**Authors:** Sven Van Segbroeck, Francisco C Santos, Ann Nowé, Jorge M Pacheco, Tom Lenaerts

**Affiliations:** 1 COMO, Vrije Universiteit Brussel, Pleinlaan 2, 1050 Brussels, Belgium; 2 IRIDIA, CoDE, Université Libre de Bruxelles, Avenue Franklin Roosevelt 50, 1050 Brussels, Belgium; 3 ATP-group, CFTC and Departamento de Fisica da Faculdade de Ciências, P-1649-003 Lisboa Codex, Portugal; 4 ML-group, Département d'Informatique, Université Libre de Bruxelles, Boulevard du Triomphe CP212, 1050 Brussels, Belgium; 5 SWITCH, VIB, Pleinlaan 2, 1050 Brussels, Belgium; 6 GADGET, Apartado 1329, 1009-001 Lisboa, Portugal

## Abstract

**Background:**

In recent years it has been found that the combination of evolutionary game theory with population structures modelled in terms of dynamical graphs, in which individuals are allowed to sever unwanted social ties while keeping the good ones, provides a viable solution to the conundrum of cooperation. It is well known that in reality individuals respond differently to disadvantageous interactions. Yet, the evolutionary mechanism determining the individuals' willingness to sever unfavourable ties remains unclear.

**Results:**

We introduce a novel way of thinking about the joint evolution of cooperation and social contacts. The struggle for survival between cooperators and defectors leads to an arms race for swiftness in adjusting social ties, based purely on a self-regarding, individual judgement. Since defectors are never able to establish social ties under mutual agreement, they break adverse ties more rapidly than cooperators, who tend to evolve stable and long-term relations. Ironically, defectors' constant search for partners to exploit leads to heterogeneous networks that improve the survivability of cooperators, compared to the traditional homogenous population assumption.

**Conclusion:**

When communities face the prisoner's dilemma, swift reaction to adverse ties evolves when competition is fierce between cooperators and defectors, providing an evolutionary basis for the necessity of individuals to adjust their social ties. Our results show how our innate resilience to change relates to mutual agreement between cooperators and how "loyalty" or persistent social ties bring along an evolutionary disadvantage, both from an individual and group perspective.

## Background

Understanding the evolution of cooperative behaviour remains one of the most exciting and fundamental challenges to date [1]. In the framework of evolutionary game theory [2,3], this problem is often analyzed using the prisoner's dilemma (**PD**) game [4]. In its popular version a cooperator is modelled as an individual that is ready to pay a cost (c) in order that another individual receives a benefit (b). A defector, on the other hand, is one who refuses to offer such help but gladly accepts b when a cooperator offers it. The accumulation of the received benefits and expended costs by all individuals, playing simultaneously as donor and receptor, is associated with the individuals' fitness and this with the individuals' social or reproductive success. Different mechanisms to promote cooperative actions in this scenario have been proposed over the years [5]. It has for instance been recognized that the population structure (who interacts with whom) plays a decisive role [6-22] (for a recent review, see also [23] and references therein). In particular, it has been shown that introducing a heterogeneous network of contacts between individuals results in an overall increase of cooperative behaviour in the most popular social dilemmas of cooperation [15,19,22]. Compared to the levels of cooperation traditionally observed in well-mixed populations [2,3], the role of heterogeneous population structures in which some individuals interact more and more often than others is impressive.

Furthermore, real life social networks share another feature that turns out to tremendously affect the viability of cooperative acts: They are examples of adaptive networks [24,25]. Our network of contacts does not remain unchanged at all times. Instead, we continuously engage in new interactions while abandoning old ones, depending on their kind. As such, individuals should not only be able to alter their behaviour, but also their social ties. The influence of this specific aspect of social networks on the evolution of cooperation has been studied by several authors [26-35].

In a minimal setting [32], individuals located at the vertices of a graph selfishly decide which social ties they want to maintain. If the individual is dissatisfied with the interaction, then she competes with her partner to rewire the link. This rewiring is done to a random neighbour of the previous partner, adding realistic spatial, social and cognitive restraints [36]. It was shown that cooperation blooms (both for strong and weak selection, see Methods) even when, on average, each individual has many social interactions, provided individuals react swiftly to adverse ties [32]. In other words, the result depends on how fast the topology is allowed to evolve. Having two processes evolving simultaneously (strategy and network structure), one parameter emerges as determinant in assessing the viability of cooperation: the ratio W = *τ*_e_/*τ*_a _of two time scales, the first associated with strategy evolution (*τ*_e_) and the second associated with evolution of population structure (*τ*_a_). High values of W reflect populations in which all their members are more apt to adapt their ties. As such, network heterogeneity and social plasticity provide a coupled mechanism that leads to both the survival of cooperation in social systems and evolved network structures that agree qualitatively with empirical analysis of real networks [37].

It is commonly observed, however, that different individuals react differently to the same situation [38,39] – some individuals have the propensity to swiftly change partner, whereas others remain connected even though they are dissatisfied with the behaviour of their partners. As such, each individual has its own innate behaviour for dealing with adverse ties and generally this innate behaviour resists change, as change brings along uncertainties. Furthermore, social networks form and evolve through individuals' decisions based on the social context wherein they find themselves: individuals, for instance, may be socially constrained not to change even when they want to. Consequently, one expects W to be an individual's characteristic and not a population attribute. As such, this strategic rewiring behaviour must co-evolve with the other features inherent to the entangled **PD **game. Making the propensity to change partners an evolutionary trait allows one to investigate how individuals should respond to adverse ties given certain social conditions defined by the underlying game. This approach opens the possibility of diversity in individual behaviours, which is ubiquitous in nature. Despite its omnipresence, it has not received much attention yet in relation to the evolution of cooperation. Only recently its role has been investigated, either as diversity in the role and position of individuals in their social network [22], diversity in individuals' game strategy [40] or in the way in which individuals change their strategy [41,42]. In the following, we combine diversity of individual context in the social network with diversity in the way individuals react to adverse social ties.

### A Minimal Model

**Figure 1:**
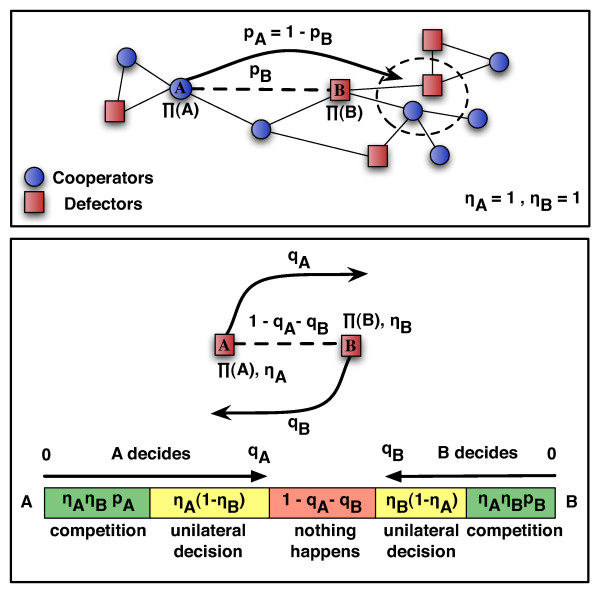
**Evolving the neighbourhood**. The upper panel illustrates the rewiring mechanism being used. A detail of a hypothetical graph is illustrated in the region surrounding the edge selected for evaluation (dashed line), connecting individuals A and B, each with a characteristic willingness to change given by *η*_A _and *η*_B_. Since A is dissatisfied, A wants to rewire whereas satisfied B will compete to keep the link. Whenever *η*_A _and *η*_B _are both equal to 1 (upper panel) rewiring takes (does not take) place with probability p_A _(p_B _= 1-p_A_), which is defined by the payoff-dependent Fermi function (see Methods) [32]. The lower panel illustrates the rewiring decisions for *η*_A _and *η*_B _in [0,1], assuming the complex situation when both individuals are dissatisfied (A and B are defectors). With probability q_A _(see below) A rewires whereas with probability q_B _it is B who rewires. With probability *η*_A _(*η*_B_) A (B) will compete to rewire the link; hence A and B compete with probability *η*_A_*η*_B_, and decision is contingent on fitness, as before (green zones); with probability (1-*η*_A_)(1-*η*_B_) no change will happen (red zone); when only one competes, decision becomes unilateral (yellow zones). Hence, A's decision prevails with probability q_A _= *η*_A_*η*_B_p_A _+ *η*_A_(1-*η*_B_), B's decision with probability q_B _= *η*_A_*η*_B_p_B _+ *η*_B_(1-*η*_A_).

Using the minimal model defined in Fig. 1, we analyze here the effect of the individual willingness to change on the evolution of cooperation in the **PD **game (see Methods). Individuals have information on their direct partners only and, using this information, they may decide to alter a link or not, based purely on their self-interest. Let us consider a link connecting individuals A and B. Two scenarios are possible: A is satisfied with the link if B is a cooperator, and dissatisfied if B is a defector. If satisfied, A will try to maintain the link. If dissatisfied, A will try to change partner (to rewire the link, Fig. 1 top panel). Decision on whether or not to rewire is contingent on both individuals' payoffs, and here determined by the Fermi function (see Methods) associated with the so-called pairwise comparison rule. In this way the model entangles the evolution of individual's strategy and social structure.

The individual rewiring rate is defined over an interval [0, W], where W reflects the common (maximum) rate of topological change that each individual can reach. Taking a particular W as a limit ensures that we know a priori the outcome of evolution [32]. Given this context, we introduce an individual characteristic *η*∈[0,1], which provides a measure of how eager each individual is to change partners, making *η*W the individuals' willingness to rewire unwanted social interactions. Thus, on the one hand, when *all *individuals have *η *= 0 no links are rewired, reducing the model to a static society. On the other hand, when the rate is maximal for *all *individuals (*η *= 1) the limits investigated in [32] are recovered. When different individuals have different values of *η*, those with lower *η *< 1 will be more resilient to change, and hence can also be viewed as more loyal towards their interacting partners. Given this particular definition of how each individual decides upon adverse ties, we can now define each individual type uniquely by two parameters: her game strategy (s) and her "topological" strategy (*η*). Note that both behavioural strategy s as well as the topological strategy *η *are transferred during a strategy update.

In this minimal model, the individual willingness *η*_A _and *η*_B _of two interacting individuals A and B, define three possible outcomes for the rewiring competition as a result of mutual dissatisfaction, as shown in Fig. 1 – bottom panel. Given each individual's willingness parameter *η*, A and B compete to rewire the link with probability *η*_A_*η*_B_. Individual fitness ultimately dictates the winner of this conflict, associated with the probability p_B _(p_A_) that B (A) replaces A (B), where the probabilities are defined by the payoff-dependent Fermi function (See Methods) [9,43]. When only A is dissatisfied, A and B compete with probability *η*_A_*η*_B _and with probability p_A _the A individual can change partner. Yet, with probability 1-p_A _(= p_B_) A and B remain connected since B is satisfied. Second, individual A (B) decides unilaterally to change partner with probability *η*_A _[1-*η*_B_] ([1-*η*_A_]*η*_B_). Hence, both A and B have the opportunity to unilaterally change partner. Taken together, A changes with probability q_A _= *η*_A_*η*_B_p_A _+ *η*_A_[1-*η*_B_] and B changes with probability q_B _= *η*_A_*η*_B_p_B _+ *η*_B_[1-*η*_A_] (Fig. 1, bottom panel). Finally, nothing happens to the link with probability (1-*η*_A_)(1-*η*_B_). This last possibility encompasses the situation in which the social tie is maintained despite, e.g., mutual dissatisfaction. Overall, *η *introduces a simple means to study the evolution of each individual's willingness to react to adverse ties.

## Results and discussion

**Figure 2:**
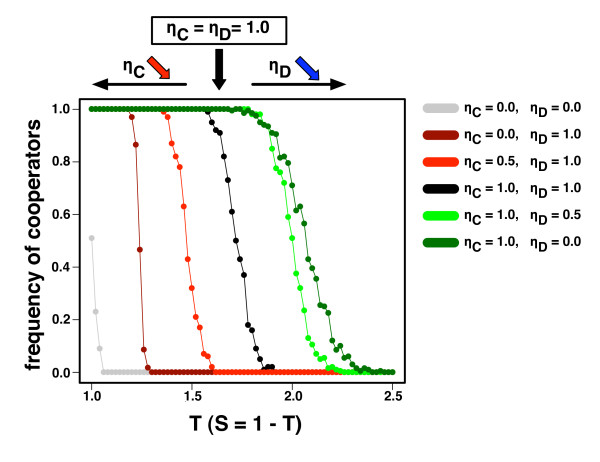
**Role of strategy-dependent willingness on cooperation**. Fraction of cooperators as a function of T for different values of *η*_C _(the willingness to change of cooperators) and *η*_D _(the corresponding quantity for defectors). The remaining parameters are W = 2.5, *β *= 0.005, N = 10^3 ^and z = 30. The situation in which *η*_C _= *η*_D _= 1.0 is here used as baseline. Reducing *η*_D _makes it easier for cooperators to wipe out defectors. Reducing *η*_C_, on the other hand, has the opposite effect.

As a first step to investigate the effect of differences in eagerness to change on the evolution of cooperation (see Methods), we assume that either cooperators or defectors have a fixed and pre-defined *η *(see Fig. 2). The chances of cooperators are measured by calculating the fraction of runs in which the population ends in full cooperation. Note that the adopted strategy update rule ensures that full cooperation and full defection are the only two absorbing states of the strategy evolutionary dynamics (see Methods). Fig. 2 shows that when defectors are less eager to change partners (*η*_D _= 0.5 and *η*_D _= 0) relative to cooperators (*η*_C _= 1.0), cooperators ensure the stability of favourable interactions while avoiding adverse ones more swiftly; hence, assortment of cooperators becomes more effective, enhancing the feasibility of cooperation [44]. When cooperators' willingness to change is low (*η*_C _= 0.5) or absent (*η*_C _= 0.0) compared to defectors (*η*_D _= 1.0), the level of cooperation decreases with respect to the situation where cooperators and defectors react equally swift to adverse ties (*η*_C _= *η*_D _= 1.0). Decreasing the level of adaptability of cooperators leads to their own demise. On the other hand, if we compare these results with those in which all social ties remain immutable (leading to a static network, *η*_C _= *η*_D _= 0, shown also in Fig. 2), the feasibility of cooperation actually increases. Why does rewiring of defector-links already improve the survival of cooperators? This latter result is a consequence of heterogeneity created by rewiring defectors. As we start from well-mixed communities of limited connectivity (see Methods), rewiring of links creates a heterogeneous environment, which always favours cooperators. Thus even when cooperators are slow adapters, they prosper at the expense of the defectors greed. Overall, our results clearly show that swift decisions concerning partner choice provide a proactive force toward the evolution of cooperation, *independent of the strategy*.

**Figure 3:**
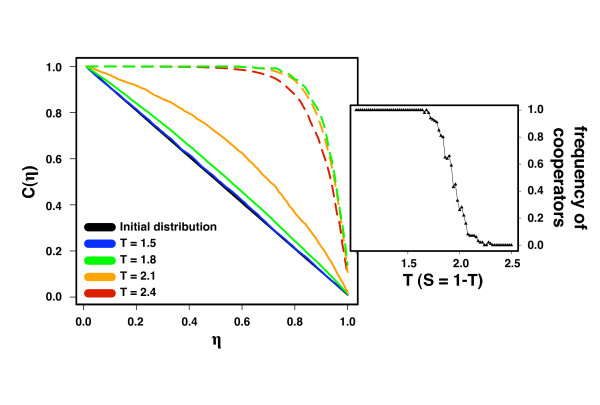
**Evolution of *η *for cooperators and defectors**. The solid (dashed) lines show the cumulative distribution C(*η*) of the parameter *η *of cooperators (defectors) for different values of T (W = 5, *β *= 0.005, N = 10^3^, z = 30). The inset provides the level of cooperation for values of T between 1 and 2.5. The values of *η *of all individuals are uniformly distributed in [0,1] at the start of each evolution, as indicated in black. Cooperators that react swiftly to adverse ties are strongly favoured by natural selection only when the tension of the game is high (1.8 ≤ T ≤ 2.1). Swift defectors, on the other hand, are always selected for any tension of the game, but the strength of this selective pressure drops as T increases. C(*η*_0_) is defined as the fraction of individuals who have *η *≥ *η*_0_.

Given this effect of topological strategy on the outcome of the **PD **game, we analyze the effects of evolving this feature as well. To this end, every time an individual changes her strategy by adopting that of a neighbour, she also changes her topological strategy to that of her neighbour. In Fig. 3, we show the evolution of the willingness to change ties of both cooperators and defectors. In both scenarios all values of *η *are selected from a uniform distribution and assigned to individuals in the population.

We analyze the distribution of *η *at the end of the evolutionary process when the population reaches fixation (all individuals adopt the same strategy). From the results discussed previously one might expect that swift action is always preferred to stubbornness. The lines in Fig. 3 correspond to the cumulative rate distribution C(*η*) (C(*η*_0_) is defined as the fraction of individuals who have *η *≥ *η*_0_) for both cooperators (solid lines) and defectors (dashed lines). The initial distributions lead to the black diagonal lines in Fig. 3; the final distributions are shown with different colours for different values of the temptation to defect T of the **PD **game (see Methods). For low values of T (< 1.8) the distribution of *η *over all individuals hardly changes. For this range of T (see also Fig. 2) cooperation prevails, and hence individuals rapidly become satisfied with all their links. For higher values (1.8 ≤ T ≤ 2.1) a transition occurs from cooperator dominance to defector dominance (as shown in the inset of Fig. 3). Hence competition is fierce, as cooperators need to struggle for survival.

Consequently, it pays to respond swiftly to adverse ties and evolution leads to an arms race for swiftness between cooperators and defectors, as evidenced by the increase of C(*η*) in Fig. 3. For even larger values of T (> 2.1) defectors dominate the results and evolutionary competition fades away. As a result, the incentive to increase swiftness reduces, a feature which is indeed reflected in the behaviour of C(*η*) in Fig. 3. Once all individuals ultimately become defectors there are no fitness differences and hence no selection pressure to further changes. Nonetheless, the fundamental differences between cooperators and defectors still have an impact in the overall evolutionary dynamics. When cooperators dominate, many social ties rely on mutual satisfaction, and hence there is no incentive to change. On the contrary, even when dominant, defectors are never able to find a partner with whom mutual satisfaction occurs, as a defector with local information only will always strive to find a cooperator to exploit, whereas a cooperator will strive to escape exploitation. Consequently, under cooperator dominance we reach a stable and slowly changing network of ties. In the opposite limit, a quasi-static network is never reached; instead, a stationary one emerges, exhibiting an intrinsic degree of heterogeneity that decreases with increasing number of defectors [32]. Besides the results discussed here, other features of the co-evolutionary dynamics are also affected by introducing a co-evolutionary dynamics in which individual swiftness in reacting to adverse ties also evolves. In particular, the characteristic times required to reach a stationary regime associated with either full defection or full cooperation depend on individual behavioural diversity. This, in turn, is intimately related to the topology of the evolving graph, which itself depends sensitively into which stationary state the system evolves. A detailed account of the timing features and how they depend on the amount of diversity as well as on the relative time scales of co-evolution will be published elsewhere.

## Conclusion

Our results clearly provide an evolutionary basis for the necessity of individuals to adjust their social ties. The struggle for survival between cooperators and defectors leads to an arms race for swiftness in adjusting the ties, based on a self-regarding judgment. Since defectors are never able to establish social ties under mutual agreement, they are overall swifter than cooperators, who tend to evolve stable and long term relations when the risk of exploitation is absent. Ironically, defectors' constant search for partners to exploit leads to heterogeneous networks that improve the survivability of cooperators compared to homogeneous populations. At the same time, swifter defectors prevent cooperators from wiping them out.

The existence of stable communities such as families, groups, political parties and other social agglomerates, relies on the persistence of social ties, being usually related to an idea of "*loyalty*" which is often associated with some form of survival advantage [45]. However, in modern networks of exchange and cooperation, where partnership preferences have already surpassed the limitations imposed by kin-like constraints, these social structures are evanescent. Populations of self-regarding individuals engage in increasingly diverse, short-lived and geographically uncorrelated social ties. In this context, our results show that "*loyalty*" or persistent social ties bring along an evolutionary disadvantage, both from an individual and a group perspective. Once the individual ability to freely reshape partnerships arose – most probably originating from the human organization into increasingly larger communities (with associated increasing return benefits [46]) – those individuals that acquired the aptness to respond quickly to unwanted relationships obtained an evolutionary edge over other individuals that remained stuck to the same social ties whatever the cost.

Consequently our results may provide some important hints on why "persistence of social ties" plays a smaller role in humanity nowadays, as cooperation based on kinship and geographical proximity becomes replaced by increasingly global and volatile exchanges of social and economical nature. Our results suggest that the rapid emergence of online social communities – such as the ones involved in wiki's or open source projects – that are mostly based on cooperative efforts while devoid of norm enforcement mechanisms, may be linked to this change of paradigm, which may ultimately provide the escape hatch from the global challenges of cooperation we also face [47-49].

## Methods

### Graphs

We assign individuals to the vertices (a fixed total of N) of a graph. The edges of the graph (a fixed total of N_E_) represent social ties between individuals. Each simulation starts with a homogeneous random graph [50] (or regular random graph), in which all vertices have the same number of edges (z = 2*N*_*E*_/*N*), randomly linked to arbitrary vertices. This configuration mimics a well-mixed population in which connectivity is limited. The degree distribution of the graph changes over time as individuals change their ties. The average connectivity z is conserved since we do not introduce or destroy any edges.

### Prisoner's dilemma

We study the well-known prisoner's dilemma (**PD**) game, in which players can either cooperate or defect during an interaction. Mutual cooperation leads to a reward R for both players, mutual defection to a punishment P. When one player cooperates while the other defects, the cooperator (defector) receives the sucker's payoff S (temptation to defect T). Depending on the relative ordering of these payoff values, different social dilemmas arise [51]. We enter the realm of the **PD **whenever T > R > P > S, the cost-benefit dilemma described in the Background section constituting the prototypical example. In that case, mutual cooperation leads to R = b-c, whereas mutual defection leads to P = 0. When a cooperator and a defector interact, the cooperator gets S = -c, whereas the defector gets T = b, automatically satisfying the inequality above. We normalize the advantage of mutual cooperation over mutual defection to 1 [19], making R = 1 and P = 0. This leaves two parameters, T and S, to tune the intensity of the social dilemma inherent to the **PD**. Together T and S define a 2D parameter space, of which we consider the diagonal defined by T (and S = 1-T). This diagonal corresponds exactly to those games that can be associated with a costbenefit parameterization of the prisoner's dilemma: With T = b and S = -c, R = 1 leads to T+S = 1.

### Evolution of strategies

Individual strategy and network structure co-evolve under asynchronous updating. The type of update event – behaviour or partner – is chosen according to the ratio W between the time scales associated with the evolution of strategy (*τ*_e_) and of structure (*τ*_a_). Assuming *τ*_e _= 1 (without loss of generality), a strategy (behavioural) update event is chosen with probability (1+ *W*)^-1^, a structure (partner) update event being selected otherwise. A strategy update event is defined by the pairwise comparison rule [9,43]: An individual A is drawn randomly from the population and another individual B is chosen at random from the immediate neighbours of A. The strategy of B will replace that of A with a probability given by the Fermi function *p*_*B *_= [1 + *e*^-*β*(Π(*B*)-Π(*A*))^]^-1^, where Π(X) represents the accumulated payoff of player X after interacting with all her neighbours. The value of *β *(≥0) controls the intensity of selection (*β *→ 0 leads to neutral drift whereas *β *→ ∞ leads to the so-called imitation dynamics, often used to model cultural evolution).

### Evolution of individual ties

At each structure update event, a random edge is selected for evaluation, as illustrated in Fig. 1. The two individuals at the extremes of that edge – A and B – decide unilaterally what they wish to do. Whenever both A and B are cooperators, both are satisfied with the edge and no rewiring takes place. The two other possibilities occur when either A and/or B are defectors, as at least one individual will necessarily be dissatisfied with the edge. These scenarios are represented schematically in Fig. 1. If both A and B are dissatisfied (or if one is satisfied while the other is not) both have the chance to compete for whatever they wish to do (keep the link if satisfied, rewire if dissatisfied). A will compete with probability *η*_A _whereas B will compete with probability *η*_B_. If both actually compete, B's decision will prevail with the probability p_B _defined above in terms of the Fermi function, whereas A's decision will prevail with probability p_A _= 1-p_B_. If only one actually competes, decision will be unilateral, whereas if no one competes nothing will happen. When decision is to rewire, the new destination is chosen randomly from the immediate neighbours of the former opponent (as illustrated in the upper panel of Fig. 1). We impose that individuals connected by a single link cannot lose this link, hence preventing the network from becoming disconnected, allowing all nodes to undergo evolution of strategies.

### Computer simulations

We use networks of size N = 10^3 ^with average connectivity z = 30. The latter value reflects the average connectivity of a plethora of empirically studied social networks [52,53], with connectivity values ranging from 2 to 170, with an associated heterogeneity intermediate between single-scale and broad-scale [52]. We start with 50% cooperators, randomly distributed in the population. The results in Fig. 2 are obtained by running 100 independent simulations for each set of parameters (T;*η*_C_,*η*_D_) and computing the fraction of times that evolution stopped at 100% cooperation. Each of the cumulative distributions of *η*, C(*η*), for cooperators (defectors) in Fig. 3 is obtained after running 10^3 ^simulations that fixate in 100% cooperation (defection) for a given value of T. At the end of each evolution, we calculate the cumulative distribution of *η *for either cooperators or defectors. C(*η*) in Fig. 3 is defined, for a given *η*_0_, as the fraction of individuals who have *η *≥ *η*_0_. The results are robust with respect to changes both in the average connectivity and in the value of W.

Note that given the stochastic nature of the pairwise comparison rule [43] adopted, the strategy evolutionary dynamics exhibits only two absorbing states, at 100% of cooperation or at 100% of defection. Whenever the cooperator absorbing boundary is reached, the co-evolutionary dynamics stops, as all social ties are established between cooperators, entailing mutual satisfaction. Whenever the full defection absorbing state is reached, only strategy dynamics will stop, as mutual dissatisfaction will compel myopic defectors to search uninterruptedly for other partners, leading ultimately to a stationary regime in which the average properties of the population structure remain unchanged.

## Authors' contributions

SVS, FCS, JMP and TL conceived and designed the experiments, performed the experiments and analyzed the data. SVS, FCS, AN, JMP and TL wrote the paper.
